# Patient and GP experiences of pathways to diagnosis of a second primary cancer: a qualitative study

**DOI:** 10.1186/s12885-021-08238-0

**Published:** 2021-05-03

**Authors:** Debbie Cavers, Rhona Duff, Annemieke Bikker, Karen Barnett, Lovney Kanguru, David Weller, David H. Brewster, Christine Campbell

**Affiliations:** 1 Usher Institute of Population Health Sciences, Medical School, University of Edinburgh, Teviot Place, Edinburgh, EH8 9AG UK; 2 Institute of Applied Health Research, University of Birmingham, Edgbaston, Birmingham, B15 2TT UK; 3 Population Health and Genomics, School of Medicine, Ninewells Hospital, University of Dundee, Dundee, DD1 9SY UK; 4 NCJDRSU, Western General Hospital, Crewe Road South, Edinburgh, EH4 2XU UK

**Keywords:** Second primary cancer, Qualitative, Pathways to diagnosis, Survivorship, Primary health care, General practice

## Abstract

**Background:**

More people are surviving a first primary cancer and experiencing a second, different cancer. However, little is known about the diagnostic journeys of patients with second primary cancer (SPC). This study explores the views of patients and general practitioners (GPs) on their experiences of pathways to diagnosis of SPC, including the influence of a previous diagnosis of cancer on symptom appraisal, help-seeking and referral decisions.

**Methods:**

Qualitative interviews with patients with a SPC diagnosis and case-linked GP interviews in a Scottish primary care setting. In-depth face to face or telephone interviews were conducted, underpinned by a social constructionist approach. Interviews were transcribed and Braun and Clarke’s thematic analysis undertaken. Three analysts from the research team read transcripts and developed the coding framework using QSR NVivo version 10, with input from a fourth researcher. Themes were developed from refined codes and interpreted in the context of existing literature and theory.

**Results:**

Interviews were conducted with 23 patients (aged 43–84 years) with a SPC diagnosis, and 7 GPs. Five patient themes were identified: *Awareness of SPC, symptom appraisal and help-seeking, pathways to diagnosis, navigating the healthcare system*, and i*mpact of SPC*. GPs interviews identified: e*xperience and knowledge of SPC* and r*eferrals and decision-making.*

**Conclusions:**

Insights into the pathway to diagnosis of SPC highlights the need for increased awareness of and vigilance for SPC among patients and healthcare providers (HCPs), and emotional support to manage the psychosocial burden.

**Supplementary Information:**

The online version contains supplementary material available at 10.1186/s12885-021-08238-0.

## Background

The number of people surviving cancer is increasing [1] and, in combination with an ageing population, the incidence of second primary cancers (SPCs) will also inevitably rise [2]. Within the context of cancer survivorship, recurrence and metastatic disease research, there is a small but growing literature on SPC [3–5]. Research on SPC to date has focused on incidence, risk (from environmental, lifestyle and genetic factors), and late treatment effects following a first primary cancer (FPC) e.g. [6–8]. The reported risk of developing SPC among cancer survivors ranges from 1 and 17% depending on the index cancer site e.g. [7–9]. A West of Scotland cancer registry study reported that among 57,393 cancer survivors, 5 % were diagnosed with SPC within 5 years of their first diagnosis [10]. SPCs are estimated to account for as much as 16–18% of total cancer incidence [11–13].

**Fig. 1:**
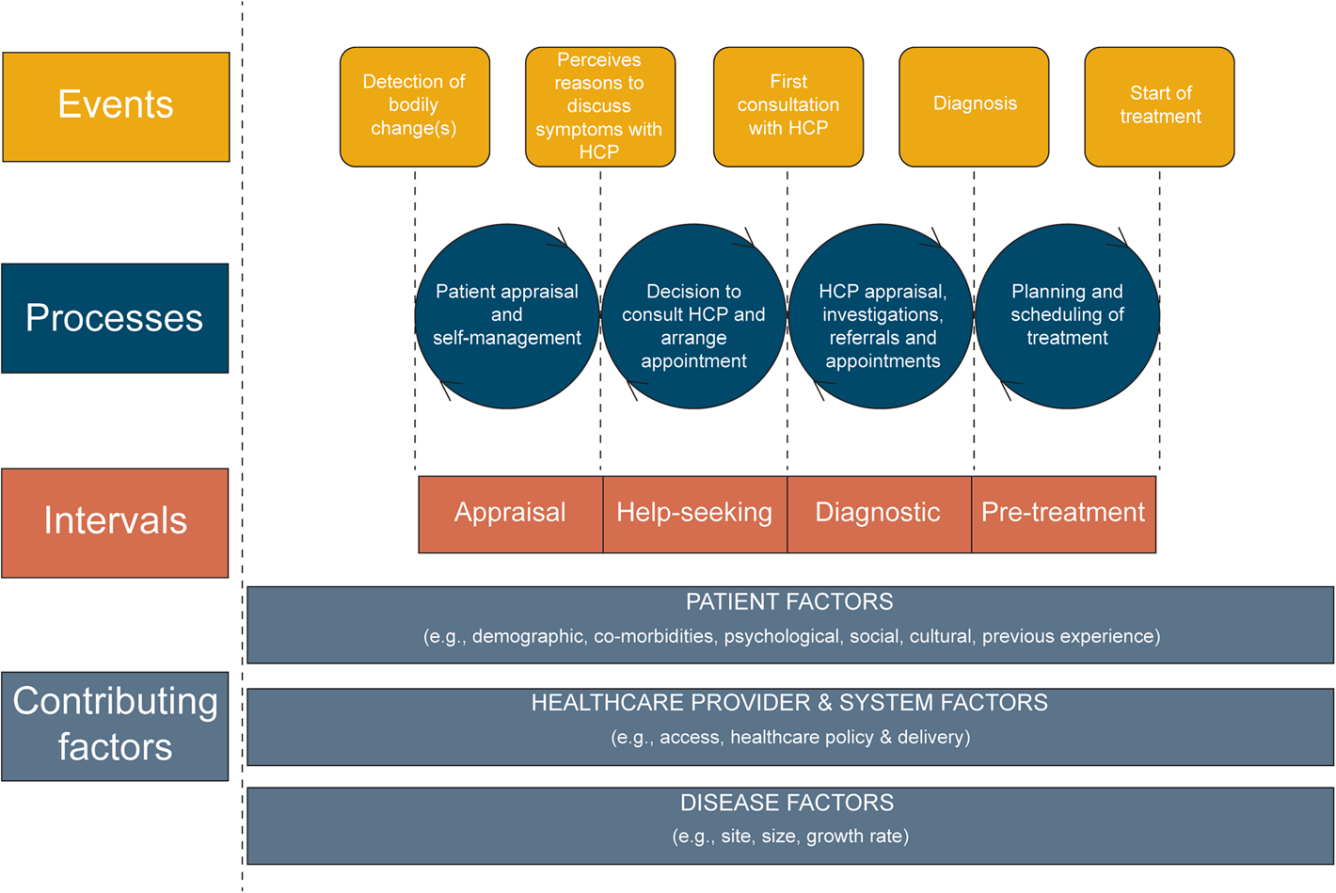
Pathways to treatment model [14]

Relatively little is known about pathways to diagnosis of SPC. The Pathways to Treatment Model (see Fig. 1) highlights the complexities of multiple patient factors - including psychological features and prior experiences - in influencing symptom appraisal and help-seeking behaviours, which may also be applicable to SPCs [14]. The limited evidence suggests that although fear of a second cancer has been identified as a source of worry and psychosocial distress [4], there is a general lack of awareness regarding risk of SPC [5]. There is a need for greater understanding of differences between diagnostic journeys for recurrence of FPC versus a second, different cancer, and their associated psychological dynamics. There are implications for awareness-raising and, if appropriate, designing behavioural interventions to facilitate early detection of SPC [4, 15].

Further, there are challenges for primary care in managing cancer follow-up, and SPCs will be an increasing feature of this care [5, 16–18]. Not enough is known about the impact on primary care of a growing burden of complex disease, including second and subsequent primary cancers [19]. Exploring the extent to which the policy drive for early detection and treatment of cancer applies to SPC in its current form is warranted [2, 20].

Understanding cancer survivors’ lived experiences of SPC and their pathways to diagnosis, as well as the perspectives of general practitioners (GPs) managing them, will contribute to the growing evidence base on best practice in cancer survivorship care.

### Aims and objectives

This study aimed to examine: 1) experiences of people diagnosed with SPC, 2) the influence of a previous experience of cancer on the SPC diagnostic pathway, including symptom appraisal and help-seeking, and 3) experiences and challenges of diagnosing SPC from the GP perspective.

## Methods

The study involved a series of in-depth interviews carried out in 2018 with patients diagnosed with SPC in Scotland, and the GPs involved in diagnosing and managing SPCs. The study was based on a social constructionist approach and conducted by a research team comprised of clinical and academic health service researchers experienced in researching pathways to diagnosis and early detection of cancer. This article has followed the COREQ reporting guidelines (see supplementary file 1).

### Participants

Participants were patients who have had a diagnosis of SPC of the four main cancer types (breast, bowel, lung and prostate) within the previous 6 months, where the SPC is defined as a new cancer in the same or a different anatomical site but with different pathology to the FPC [21]. Interviews were also sought with patients’ main GPs.

### Recruitment and sampling

**Fig. 2:**
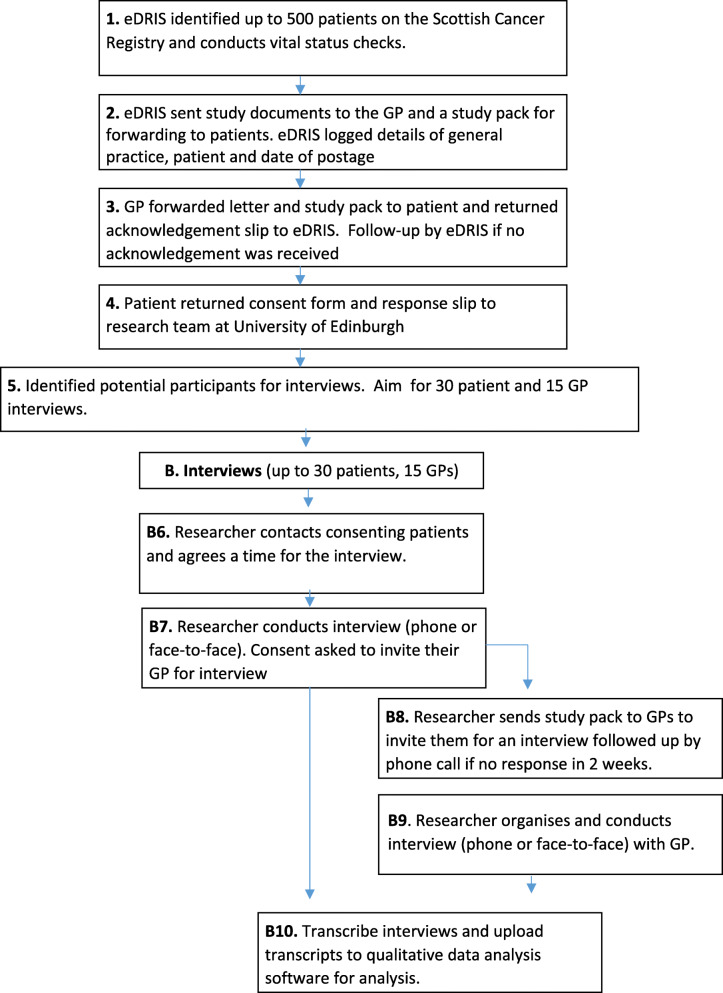
Patient and GP recruitment process

Patients were identified from the Scottish Cancer Registry, by NHS staff working for National Services Scotland’s Electronic Data Research and Innovation Service (eDRIS). Recruitment packs (information sheet, consent form and reply slip) were sent with a covering letter to eligible patients’ GPs to confirm diagnosis and screen for suitability. To protect patient confidentiality, GPs were asked to forward the packs to appropriate patients. Interested patients returned a reply slip and consent form to the researcher (AB, an experienced health services and qualitative researcher with a background in social anthropology) who contacted them to build rapport, discuss the study, and arrange an interview. Due to challenges in recruiting this select group, all eligible patients who responded were interviewed. However, the participants were diverse in terms of gender, location and cancer types. No one withdrew from the study. With patient consent, GPs were then invited for interview (see Fig. 2 - recruitment process).

### Interviews

Interviews were one-off and semi-structured, using open-ended questions and a patient-centred approach to explore patient experiences of both first and second primary cancer, including psychosocial dimensions of experience and pathways to diagnosis. The topic guide was developed (for this study) in the first instance by AB and refined with input from DC, CC and a patient representative, using their experience of interviewing on the topic of pathway to diagnosis and living with cancer as well as a patient perspective (see supplementary file 2). Written consent was given prior to, and checked before, the interview commencing. Interviews were face to face in patients’ own homes, in a university meeting room, or by telephone, lasting approximately 1 h. Participants were aware of the objective of the study and that AB was a health services researcher from a non-medical background. Field notes were taken and used to inform subsequent analysis and aid reflexivity.

Face to face and telephone interviews were carried out with case-linked GPs (including specific discussion of the patient’s case if relevant).

### Analysis

All interviews were digitally recorded, transcribed and anonymised before being uploaded to QSR Nvivo version 10 (www.qsrinternational.com). Data were subject to Braun and Clarke’s thematic analysis [22]. Transcripts were read by three researchers (RD, DC and CC with additional input from AB, thus ensuring triangulation and rigour in the analytic approach), and re-read for the purposes of familiarisation; looking for emergent and recurrent concepts. Appraisal across data compared and contrasted to establish common codes. Codes were then applied back across the data to ensure that no new codes or concepts were apparent, such that a level of data saturation was reached. In addition, through an iterative process of analysis of patient interviews, no new themes were apparent with subsequent interviews, suggesting a sufficient sample size for thematic saturation, although we acknowledge the limitations of this notion and recognise that the concept of saturation has been problematised in the literature [23]. Limitations of recruitment to GP interviews and subsequent saturation are expanded on in the discussion. Any deviation from core themes was explored and interpreted by the research team and consensus was reached among analysts. Findings were considered, developed and reported as themes in the context of existing theory and research, including the Pathways to Treatment model. Due to time and other resource constraints, a summary of the analysis was not returned to participants for member checking.

## Results

### Patient interviews

Of 23 people interviewed, 20 met the final inclusion criteria (see Table 1); 15 women and five men, ranging in age from 43 to 84 years. Time between first and second diagnoses ranged from 1 to 34 years. Ten participants were diagnosed by presenting to primary care with symptom concerns, while the remainder had their cancers detected incidentally, through screening or during specialist follow-up for their FPC. All participants had at least one form of comorbidity.

**Table 1:** Patient demographics and characteristics of FPC and SPC

	Sex/ Age range	FPC Diagnosis	Mode of Detection	Time Interval FPC /SPC	SPC Diagnosis	Mode of Detection	Self-Reported Co-morbidities/ Treatment Complications
**1**^**★**^	M, 70–74	Bowel	GP consult	20 years	Prostate	Incidental	Atrial fibrillation, B12 deficiency
**2**	F / 65–69	Bowel	Incidental	19 years	Breast	Nurse Consult	Ileostomy problems for 7 years+, *C. difficile*
**3**	F / 40–44	Cervical	Screening	15 years	Breast	GP consult	RTx damage to bowel, early menopause
**4**	M / 60–64	Kidney	GP consult	24 years	Prostate	GP consult	Cirrhosis, *E. coli*
**5**	F / 60–64	Melanoma	GP consult	16 years	Bowel	GP consult	High blood pressure
**6**	F / 70–74	Breast	GP consult	3 years	Bowel	Screening	High blood pressure, seroma
**7(8)**	F / 70–74	Breast	Incidental	34 years	Bowel	GP consult	Peripheral neuropathy, asthma
**8(9)**	F / 65–69	Pancreatic	GP consult	10 years	Breast	Screening	Chronic fatigue syndrome, fibromyalgia
**9(11)**	F / 70–74	Breast	Screening	7 years	Lung	GP non-cancer	Osteoporosis
**10(13)**	F / 60–64	Endometrial	GP consult	20 years	Colorectal	GP consult	RTx damage, bladder problems, MRSA
**11(14)**	F / 60–64	Lymphoma	GP consult	9 years	Lung	Incidental – via FPC	Arthritis
**12(15)**	M / 70–74	Larnyx	Incidental	3 years	Prostate	GP consult	Back pain
**13(16)**	M / 75–79	Prostate	Diabetes check-up	1 year	Lung	Incidental – via FPC	Diabetes, Macular degeneration
**14(17)**	F / 75–79	NHL	Incidental	5 years	Bowel	Secondary care consult	Heart condition
**15(18)**	F / 80–84	NHL	Emergency	5 years	Breast	Breast clinic	COPD
**16(19)**	F / 60–64	Breast	Screening	6 years	Colon	GP consult	Underactive thyroid, peripheral neuropathy
**17(20)**	F / 60–64	Melanoma	GP consult	21 years	Breast	Screening	Restless leg syndrome, IBS, depression
**18(21)**	F / 45–49	Lymphoma	GP consult	8 years	Breast	GP consult	Early menopause
**19(22)**	M / 65–69	Bladder	GP consult	1 year	Prostate	Incidental – via FPC	Diabetes, high blood pressure, high cholesterol
**20(23)**	F / 50–54	Melanoma	GP consult	22 years	Breast, then Thyroid	Incidental	Thyroid problems

^**★**^Of 23 people interviewed, 20 met the final inclusion criteria

A number of central themes evident in the data are reported. Additional supporting quotes for some themes relating to patient interviews can be found in Table 2.

**Table 2:** Patient interviews: additional quotes

Themes	Supporting quotes
*Awareness of SPC*	*“Interviewer: Did anybody ever mention to you about the risk of a second primary cancer or was it ever on your mind…?* *P16: Never, no, no, I didn’t think at all.* “***P13*** *“I just completely forgot. […], It’s because of having no problems with the...after the first one that I just forgot all about it, I really did.”* ***P14***
*Symptom appraisal and help-seeking*	*“…Very tired, […] I’m sitting at nine o’clock sleeping, and I thought this is not normal. So, I went to see my GP and they took blood tests and then sent me for an X-ray, and that’s when they discovered that I had a tumour in my lung.”* ***P1*** *“I’m probably less inclined to just sit on* *something and not go to the GP, as a result.”* ***P18***
*Pathways to diagnosis of SPC*	*“I will be absolutely frank, I never thought, oh goodness, that’s the second time I’ve had cancer […].[…]. That wasn’t the first thing that came to my mind. I would say it took me quite a long time to kind of clock that actually, that means I’ve had cancer twice. It just wasn’t top of my list of priorities to be perfectly honest.”* ***P5***
*Navigating the healthcare system*	*“I go to that urology clinic, it’s packed, it’s absolutely mobbed. It’s a really busy place […]. When I went to the kidney clinic, when I first got it, there was only me, you know, there wasn’t a big queue or nothing.”* ***P4***
*Impact of SPC*	*“Interviewer: Have they ever mentioned your first cancer, the melanoma or was that kind of never discussed?* *P5: Not really discussed, I mean they knew, but it wasn’t linked and nobody kind of majored on it****.****”* ***P5***
*Cumulative burden of SPC*	*“I thought, oh, no, here we go again.”* ***P8***

#### Awareness of SPC

Overall, there was low awareness among patients about the risk of developing SPC. SPC diagnosis came as a big shock to many,

*“It was totally unexpected because I didn’t realise that you could have two cancers running at the same time.”* ***P11***

Patients were more likely to fear recurrence of their FPC. In some cases, however, there had been such a long interval between their diagnoses that their FPC had receded in significance or, in one case, they had forgotten about it altogether.

SPC was not something that was reportedly discussed with their GP or oncologist during treatment or follow-up of the FPC and, on the whole, participants did not think it influenced the timeliness of their referral for their SPC (something that was echoed in GP interviews).

#### Symptom appraisal and help-seeking

Pathways to diagnosis described by participants have a marked similarity to those of a FPC.

In the case of those who had symptoms, participants reported that they responded to these by telling their GP or FPC consultant very quickly,

*“..If this was a completely isolated, never had breast cancer before, I think I would have reacted the same way. I think I would have had that feeling of, something’s not quite right here.”* ***P16***

Participants also reported similar barriers and facilitators to those reported elsewhere in relation to pathways to diagnosis of a FPC, including issues such as normalising symptoms (e.g. attributing them to age or other ongoing comorbidities), work demands, masculine identity, and being encouraged by family members to seek help,

*“But, men don’t bother as much as, […] I don’t think.”* ***P1***

There is a suggestion in the data that the help-seeking interval in the pathway to diagnosis may have been expedited as a result of their previous cancer. A number of participants reported, on reflection, a quick and sometimes urgent response in seeking advice from a HCP because of their previous cancer diagnosis,

*“The last thing with my history is to leave it.”* **P2**

Similarly, some participants could have had their help-seeking and diagnostic intervals expedited by seeking help when at an FPC appointment,

*“I noticed the lump. And I was due to see the nurse a few days later to take bloods. And I said to her, look what do you think? And she said, I think you should be seeing the doctor. […] It was a doctor I’d never met before […] But she said, right I’m referring you straight to the breast clinic at the [HOSPITAL].”* ***P2***

And while overall participants suggest that first primary cancers were not taken into consideration, some participants perceived that it did influence their care second time around,

*“I think because I’ve had cancer before that when I get appointments in the hospital they’re pretty quick.”* ***P4***

#### Pathways to diagnosis of SPC

Routes to diagnosis varied among participants, with roughly half of diagnoses as a result of a GP referral following primary care attendance (see ‘Mode of Detection’, Table 1). The remaining half were picked up via screening or in secondary care whilst attending for follow-up of the FPC, both in the presence and absence of symptoms.

Participants could be roughly divided into two groups in the lead up to their SPC diagnoses. The first describe a fairly direct and uncomplicated path between one cancer diagnosis and another, often with many years between diagnoses and no lasting effects of their FPC following successful treatment,

*“So I reacted quite quickly and I got an appointment with my GP and my GP was excellent. They did an internal […] and she sent me for an urgent colonoscopy and I got that the following week.”* ***P16***

For this group, the first cancer is reported as something in the past that was treated and doesn’t have a lasting physical impact on their lives. The second cancer has come as a shock and was not expected.

For the second group of patients, the route between diagnoses was less straightforward and people were often dealing with recurrences, long-term side effects of first cancer treatment (e.g. stoma) or significantly disabling comorbid conditions,

*“I didn’t have time to worry about it ‘cause it was always… my ileostomy [as a result of first primary bowel cancer] that takes over. And I have to just live my life to keep that working properly without worrying whether my breast cancer is going to come back again or not.”* **P2**

This group of patients is more likely to be in regular contact with healthcare services for ongoing treatment and review. While still unexpected and unwanted, the impact of the SPC can be considered to have had less of an impact on already turbulent circumstances.

#### Navigating the healthcare system

Previous experience of cancer did mean that participants knew, to a degree, what to expect and, in many cases, found the practical element easier second time around. People were more familiar with the process and navigating the health care system,

*“But as I say, I think because of previous experiences and knowing the system and everything, I was able to just rationalise it pretty quickly and say, this is stupid. […] I could apply colorectal to the problem, but obviously it wasn’t exactly the same. But it gave me, sort of, insight and, you know…so that I wasn’t so worried […].”* ***P2***

Respondents also observed changes in health care services, contrasting their two experiences of cancer, particularly when a long time had elapsed between diagnoses. While acknowledging that treatment and technological advances had been made, people perceived a busier, resource-constrained environment, with more pressure on staff to meet demands. They described crowded waiting rooms, longer waiting times and difficulties getting appointments in primary care,

*“I feel really disappointed […] I think it’s just changing times, isn’t it? […] When I was going through all the pancreas things, for a long time after, my GP was amazing. She insisted I went to see her. […] This GP I’ve got now hasn’t even acknowledged that I’ve had breast cancer.”* ***P8***

While participants praised the NHS and the quality of interpersonal care, they suggested that overall quality of care has suffered as a result of the demand on services, harking back to a time when there was better communication and good continuity of care.

#### Impact of SPC

The impact of a second primary cancer varied according to time between and since diagnoses; type, stage and severity of cancer; symptoms experienced; and treatment and side effects. The impact of an early stage cancer treated through surgery alone was less than that of chemotherapy and radiotherapy, and with long term consequences of treatment. For participant 14, not having to go through chemotherapy for his second cancer lessened the impact on his life considerably, despite having to live with a stoma,

*“They operated the next day, and they took out a tumour which turned out to be a Dukes’ A. […] I convalesced for about two weeks and then I went back to work. […] It was just straightforward, in for the operation, and I had a stoma, and it wasn’t too bad, you know. It wasn’t too bad because I never had chemo, this time.”* ***P14***

The perceived relevance of FPC on SPC diagnosis varied between patients. Patients reported viewing their cancer diagnoses as separate events, interpreting their relevance to each other in the biological sense of having cancer in two different anatomical areas. However, the emotional relevance of each diagnosis was discussed,

*“No, they’re separate events, definitely. I definitely feel they’re separate events but I still feel…I still worry about the pancreatic cancer. That’s never, ever left me, that worrying about that. Yes, I would say I am worried about the breast cancer but, yes, it’s two different worries, isn’t it?”* **P8**

Most patients report no mention of their previous cancer diagnosis by HCPs during their SPC journey; the patients were often the first to raise the issue.

However, the impact on daily life differed from considerations of the psychological impact of having cancer twice, which could not always be accounted for in the same way.

##### Cumulative emotional burden of a SPC

In some respects, the emotional experience of a second cancer was similar to that of the first: the shock of diagnosis, struggling to make sense of what was happening, fear of recurrence and associated distress and worry,

*“No, it’s still just a massive blow when you’re told and you still go through the same shock, horror, why me, and surely this can’t be happening, not for a second time.”* ***P15***

Participants appeared to use similar approaches to coping and adjustment through narratives of luck, hope and upward social comparison (*“Other people are dying with it so I am lucky in a way”* ***P4***). However, there is evidence that going through cancer a second time added to the emotional burden of distress, worry and fear related to a cancer diagnosis.

*“I thought, you know, you’re quite blasé about it, you’re not expecting to get a second one. That really takes the wind from you. […] Quite devastating.”* ***P10***

The findings suggest that previous experience of cancer heightens the long term emotional impact and amplifies the fear of recurrence.

*“It does make you more aware of everything, you know. […] It does…which I wasn’t a worried person before, and it does make me more, everything now, oh gosh, what’s this, you know.”* **P6**

It is also important to consider the emotional and psychological impact on family members in addition to the patients themselves. This is something that was raised by a number of participants,

*“I try to protect them from it because I felt that they’d had enough. Yes, in some ways I feel like it’s been…if this had been the first time…this second cancer had been the first time I had had cancer, I think that everybody’s attitudes would be a lot different.”* **P8**

##### Fear of a third primary cancer

A unique concern that builds on fear of recurrence commonly experienced by people who have experienced SPC is the fear of a third primary cancer. A number of participants voiced worries that if they could be unlucky enough to have cancer twice, they were aware that it could happen a third time, adding to their distress and anxiety,

*“No. I mean, it’s made me think about it now, now that I know, you know, I’m maybe a bit paranoid if you like, oh, I’ve had it twice, is it going to come back again, you know.”* ***P17***

### GP interviews

Seven GPs were interviewed, coming from a range of practice sizes and locations, including one female and six male GPs, and with varying levels of practice experience ranging from 3 to 25 years (see Table 3). The key findings from the GP interviews relate to awareness of and detection of SPC as reported below.

**Table 3:** GP Characteristics

GP identifier	Years’ experience	Health Board/ relevant practice characteristics
**GP 1**	**Under 5 years**	**Lothian, large practice, 6 GPs**
**GP 2**	**21–25 years**	**Tayside, large urban practice**
**GP 3**	**Unknown**	**Lothian, large semi-rural practice**
**GP 4**	**16–20 years**	**Lothian, small, semi-rural, some deprivation.**
**GP 5**	**21–25 years**	**Lothian, urban practice**
**GP 6**	**6–10 years**	**Tayside, small rural practice**
**GP 7**	**31–35 years**	**Borders, mid-size practice, semi-rural**

#### Experience and knowledge of SPC

GPs interviewed perceived SPCs as very rare and described never before or rarely encountering them in their clinical practice. Their focus was more likely to be on recurrence of an FPC,

*“First we would probably think, is this a recurrence of the primary cancer [..], rather than a new diagnosis of another type of cancer.”* ***GP6, 6–10 years’ experience***

GPs reported not always knowing about the previous cancer. Time constraints, lack of continuity of care and time since the previous diagnosis often meant the FPC wasn’t coded on their electronic record or the GP didn’t have time to read it prior to consultation. While GPs felt they should know, it wasn’t always the case, and the onus was often on the patient to bring it up.

*“I would say it’s very rare. I mean we might not even identify that they’ve had a previous cancer; it might not be relevant.”* ***GP 1, Under 5 years’ experience***

GPs reported that SPCs were not something they generally thought about (although time between diagnoses influenced this), or discussed the relative risk of with their patients. One GP considered that it was something they perhaps should be more aware of,

*“It’s just a little lightbulb moment for everybody and, I guess, raising awareness of it... if nobody says anything out loud then it maybe just doesn’t occur to a lot of us.”* ***GP2, 21–25 years’ experience***

#### Decision-making and referrals

Similar to the findings from patient interviews, GPs described their decision-making around suspicion of cancer and referral as very similar to those of a first cancer. As with patient reports, SPCs are considered as unrelated to first primaries. GPs report that they respond to symptoms (particularly red flag symptoms) and make referral decisions accordingly,

*“I mean hopefully we refer people all fairly urgently”* ***GP 7, 31–35 years’ experience***

*“So it’s more decided on presenting symptoms rather than what happened in the past.”* ***GP3, unknown experience***

However, one GP discusses individual variation and the distinction between biological and psychosocial impact of experiencing cancer for a second time; thus recognising the importance of acknowledging previous experience of cancer,

*“I mean I think the two are probably related; I think they have the same sort of impact upon people’s kind of psyche and things. So I mean I wouldn’t necessarily associate the causality between the two of them, but I think from the patient’s perspective, they’ve got cancer, it doesn’t really matter whether it’s one or two.”* ***GP5, 21–25 years’ experience***

The same issues arise as around the challenges of diagnosing first cancers in primary care, particularly when patients are frequent attenders or have multi-morbidity clouding their cancer symptoms and in a resource constrained environment with competing priorities for patient care. Access to investigations and secondary care were also prevailing issues among interviewees.

## Discussion

### Summary of findings

This study reports the views and experiences of patients with SPC and their GPs, examining whether having previous first-hand experience of cancer influences one’s subsequent pathway to diagnosis. Patient participants reported that they hadn’t thought about getting a second, different cancer. Their symptom appraisal and help-seeking patterns were very similar to those for FPC, but with some suggestion that having a previous cancer did influence their decision-making. Patient experiences were varied, reflecting different cancer types, stages and severity of disease, and time between diagnoses. People had their second cancer detected in both primary and secondary care, both in response to and in the absence of symptoms, prompted by their help-seeking or an incidental finding. These incidental diagnoses are of interest, considering these cancers may not otherwise have been detected at this time and it is possible patients may not have consulted with symptoms, lengthening the patient interval prior to referral and diagnosis. However, participants do not explicitly refer to whether they felt being ‘in the system’ for follow-up made a difference.

Previous practical experience of navigating the health care system appeared to lessen anxiety as a result of familiarity. However, the psychological burden of SPC appeared to have a cumulative effect on people’s emotional responses, especially where treatment was more invasive and had a bigger impact on people’s lives, including those of loved ones. Moreover, knowledge of SPC raised concerns of the risk of third primary cancer, despite this risk being minimal.

GP accounts of diagnosing second cancers echo those of patients in reporting low awareness and a similar response to that of symptoms of FPC. GPs stated that they did not think of SPCs before receiving the study pack and were more vigilant for recurrence than SPC. However, they responded to red flag symptoms in the same way as they would for FPC. SPC detection is one challenge of many in the current climate of primary care.

### Comparison with existing literature and theory

Few qualitative studies report experiences of SPC. Shin and colleagues in Korea have explored patient and oncologist perspectives on SPC surveillance and describe very similar narratives on rarity and lack of awareness [5, 24]. However, past experience is likely to be more embodied and the influence of a previous cancer on people’s behaviour more complex to tease out.

There are a number of barriers and facilitators to consider in terms of factors influencing help-seeking behaviour on the pathway to diagnosis. Previous experience has been highlighted as a factor influencing symptom appraisal and help-seeking in the Pathways to Treatment model [14]. Having prior, personal, experience of cancer appears to emphasise the role of this factor. Participants in our study may be more aware of cancer and early detection as a result of their treatment and conversations with specialists, as well as public health campaigns. However, fear of the known and resulting avoidance has been identified as a barrier to help-seeking elsewhere in the literature [25]. Nevertheless, patients in this study did allude to the fact that they were less likely to ‘sit on things’ due to their past experiences. It is worth noting that this participant group were people who had mostly been successfully treated for a previous cancer and most did not have advanced disease; responding more quickly to symptoms may therefore not apply to those with more advanced illness. Previous experience of cancer and moving through the healthcare system may impact on speed and likelihood of responding to symptoms, and there is some evidence in our data to support this claim, but more detailed exploration of this aspect of health behaviour is required. We would suggest that findings on diagnostic intervals be interpreted in the context of this group of interviewees and transferability to the average patient with SPC should be done with caution. Further large scale data is needed to compare diagnostic intervals between first and second and subsequent primary cancers.

The role of procedural knowledge (gained through experience of navigating the health care system) in lessening distress resonates with the information literature e.g. [26]. Having acquired general knowledge of how departments work and what to expect from surgery and recovery through past experience, albeit for a different cancer, embeds information and removes the uncertainty associated with anxiety and distress [27]. While past experience does not appear to wholly take away the shock and distress of SPC diagnosis, it may moderate it.

GP interviews suggest that their suspicions of cancer are no different for second primaries than for first cancers. They report responding to alarm symptoms as the primary indicator prompting referral for investigations, similar to other studies focused on early detection in primary care [28]. The role of a previous cancer appears to be minimal in the present study; yet it has been highlighted as key, along with presenting symptoms, in increasing suspicion of cancer in a survey with Norwegian GPs [29]. GPs discussed the challenges of diagnosing cancer in primary care - such as lack of resources, vague symptoms and comorbidities – as described in other studies [30, 31]. However, in contrast to other studies, patients in this study did not report long delays and multiple consultations prior to second diagnosis [32], with potential implications for patient outcomes [33]. The focus on reported symptoms suggests that current research and interventions to understand, improve and support GP decision-making and referrals, as well as tackling psychological barriers to patient access to healthcare, are also relevant to early detection of SPC, as reported elsewhere [34–38]. These findings suggest that previous first-hand experience of cancer has a nuanced role in the decision-making process and there is scope for its addition to existing models of patient behaviour.

Cancer, whether it be a first or second diagnosis, appears to have a greater impact for participants if it brings more symptoms, more invasive treatment and limitations on functionality as a result of the lasting effects of surgery, chemotherapy and radiotherapy. These are the hallmarks of the cancer journey and embed cancer patienthood more forcefully, with a greater degree of disruption to daily life and personal identity or ‘biographical disruption’, for the patient and their family, and the quest for a new normal [29, 39–41]. The level of disruption, when also hit with a second cancer and potentially comorbid conditions as well, is heightened, though there may be scope for ‘procedural knowledge’ to moderate distress.

### Implications for research and practice

There are implications, therefore, for people’s ability to process the cumulative emotional burden and adjust to their illness and personal identity. Patients and GPs reported that previous cancers did not feature in discussions of their most recent cancer, but the emotional impact of previous experience was pronounced. While a previous cancer may not be significant in terms of diagnosing subsequent cancers (apart from known treatment-related or genetic links), it is clear that acknowledgement and discussion of past experiences and future risks holds psychological significance for patients’ subsequent coping and adjustment [3]. It is necessary, therefore, to consider the role of this conversation in survivorship care provided in primary care spaces [15, 17, 18].

We recommend the following: The risks of SPC should be sensitively communicated to cancer survivors; psychological support should be offered alongside conversations of risk and fear of recurrence to manage the emotional impact of a second primary cancer for patients and their families; SPC can be incorporated into the ‘past experience’ dimension of the Pathways to Treatment model to understand the journey to diagnosis of SPC; and existing efforts to detect cancer at an early stage and improve patient outcomes can be applied to SPC in their current form.

### Strengths and limitations of the study

This study provides unique insights to the experiences of people who have been diagnosed with cancer for a second time, and for the GPs diagnosing them. This rich data can help to unpick the behavioural aspects of the pathway to diagnosis of SPC, to complement the body of evidence presenting incidence, risk and survival. This study provides some interesting findings to a group of early detection researchers expecting to find differences in the way patients and professionals respond to symptoms of an SPC. In fact, the pathways are very similar, providing important evidence for early diagnosis research.

Patient interviews come from a self-selecting group of invited participants who may have above-average health; long-term survivors are more likely to be recruited in studies such as this. While this study provides important evidence that is transferable to those who have survived a first cancer and are doing well, we may be missing the voice of those with advanced and end of life disease, for whom the pathways to diagnosis may be very different and/or difficult. Participants’ experiences are also captured relatively soon after diagnosis and so a longitudinal design may have offered different insights over time. Finally, this interview study is conducted with those who self-selected to take part and therefore findings cannot be assumed to be transferable to the broader population of cancer survivors; caution in interpreting the results is warranted. Abel and colleagues have explored characteristics of responders and the role of non-response in UK-wide cancer patient experience survey [42].

Recruitment of case-linked GP interviews proved challenging and resource issues resulted in a small number of GP interviews being achieved. While worthwhile findings with strong conformity have been obtained, findings are limited in their transferability and should be interpreted in the wider context of early diagnosis and primary care research.

## Conclusions

Awareness of the risk of second primary cancer is low among cancer patients and GPs involved in cancer surveillance. However, synchronous reports from patients and GPs about symptom appraisal, help-seeking and referral for suspicions of cancer being very similar for first and second primary cancers suggest that current efforts to expedite diagnosis of cancer are as applicable to SPC as to FPC. Evidence to suggest a cumulative psychological burden of second primary cancer and fear of third primary highlight the need for past experiences of cancer to be part of the conversation and risks of subsequent cancers sensitively discussed.

## Supplementary Information

**Additional file 1.**

**Additional file 2.**

## Data Availability

Data has not been deposited in a public repository. Anonymised data is available on reasonable request to the authors.

## Abbreviations

NCJDRSU: National Creutzfeldt-Jakob Disease Research and Surveillance Unit
eDRIS: Electronic Data Research and Innovation Service
NHS: National Health Service
GP: General Practitioner
FPC: First Primary Cancer
SPC: Second Primary Cancer
HCP: Healthcare provider
