# Supplementary material for: Patient and GP experiences of pathways to diagnosis of a second primary cancer: a qualitative study
Source: BMC Cancer. 2021 May 3;21:496. doi: 10.1186/s12885-021-08238-0 (PMC8094599; doi:10.1186/s12885-021-08238-0)
Supplement: Supplementary file 2 — Additional file 2. [file 12885_2021_8238_MOESM2_ESM.docx]

**Experiencing cancer for the second time**

Interview topic guide for patient interviews

**Introduction**

The aim of the interview is to learn from you about how having had cancer before has affected your experience this time. The interview will also focus on the contact you had with your doctor or the hospital so that we can improve care for people in the future.

The interview will be about one hour and if you agree then the interview will be recorded. The interview is voluntary and you can stop or pause the interview at any time. Would you like to ask anything before we start?

**Context of their prior cancer experience and survivorship**

- Could you please tell me more about……..
- Type of first cancer
- Fear of recurrence
- Lifestyle and health behaviours (e.g. GP attendance, screening)

**Pathway to diagnosis of an SPC**

1. Symptom appraisal

- Could you please describe when and how you first noticed a symptom or a change in your body?
- What were the nature of symptoms (e.g. common, nonspecific, multiple or chronic)?
- When/why did you think that you wanted to discuss the symptom(s) with your GP?
- How was it different or similar in comparison to the previous time?
  - Views on the SPC in relation to the FPC (perceived differently or analogous/recurrence, and why)

1. Date of first presentation

- Can you please tell me about when you first went to see your GP/ went to the hospital with a symptom or concern?
  - Decision to go to the GP/hospital
  - Investigation/ referral process
  - What happened next?
  - When was the diagnosis received?

1. Views on other morbidities in relation to SPC

- Do you have any other long term illness?

**Closing**

- Could I please the year you were born?
- Do you have any other comments or views that you may feel are relevant to bring up on this topic?
- Do you have any questions?
